# Supplementary material for: Mime-seq 2.0: a method to sequence microRNAs from specific mouse cell types
Source: EMBO J. 2024 Apr 30;43(12):10. doi: 10.1038/s44318-024-00102-8 (PMC11183118; doi:10.1038/s44318-024-00102-8)
Supplement: Supplementary file 1 — Appendix [file 44318_2024_102_MOESM1_ESM.pdf]

Appendix

Appendix Fig. S1 – Processing details for small RNA libraries. *2*
Appendix Fig. S2 - Filter statistics - small RNA pipeline example B cell libraries. *3*

A Structure small RNA ligated to 3’ and 5’ linker

5’-ACACUCUUUCCCUACACGACGCUCUUCCGAUCU**NNNN**-(sRNA)$_{18\text{-}30nt}$-**NNNNNNXXXXX**AGATCGGAAGAGCACACGTCTddC-3’

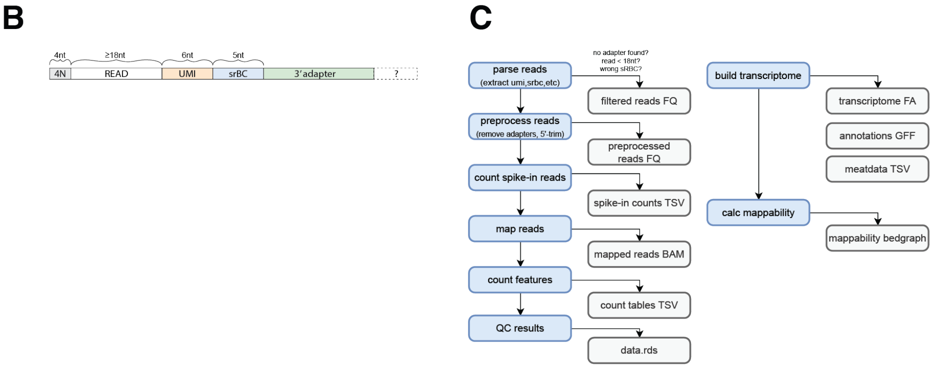

Appendix Fig. S1 – Processing details for small RNA libraries.
A. Structure of small RNA ligated to 3’ and 5’ linkers. Ns indicate random nucleotides as part of the linkers to counteract ligation bias. XXXXX indicates position of 3’ srBC barcode.
B. Outline of sequencing read structure.
C. Block diagram providing brief overview of analysis pipeline stages.

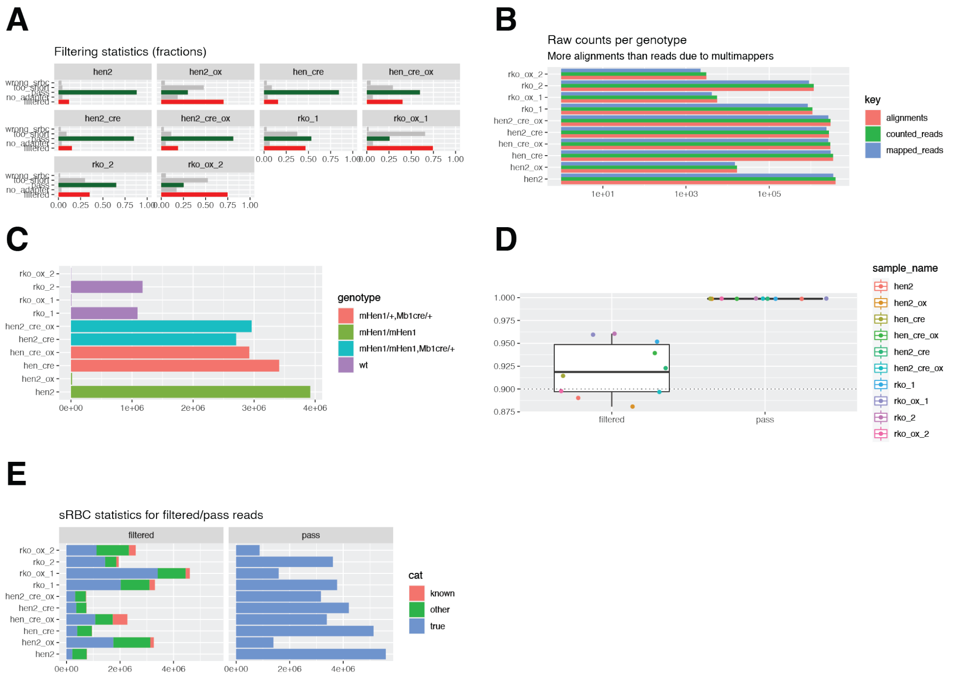

Appendix Fig. S2 - Filter statistics - small RNA pipeline example B cell libraries.
A-E For each genotype oxidized and unoxidized sample is shown.
A. Fraction of reads per sample that passed (green) or failed (red) the filtering criteria. Gray bars indicate fractions of filter reasons.
B. Number of mapped/counted reads/alignments per sample. X-axis is logarithmic.
C. Mapped read counts per sample, colored by genotype.
D. Mean adapter alignment scores for reads that passed/failed the filtering criteria; dashed line indicates the configured minimum alignment score threshold.
E. Numbers of found srBC sequences for filtered/passed reads. ‘True’: found sRBC matches expected one; ‘Known’: found srBC matches one expected for another sample; ‘other’: found sRBC matches unknown sequence. High numbers of ‘known’ sRBC sequences could reveal sample mix-ups or cross-sample contamination.
