# Supplementary figures and images for: Mime-seq 2.0: a method to sequence microRNAs from specific mouse cell types
Source: EMBO J. 2024 Apr 30;43(12):10. doi: 10.1038/s44318-024-00102-8 (PMC11183118; doi:10.1038/s44318-024-00102-8)

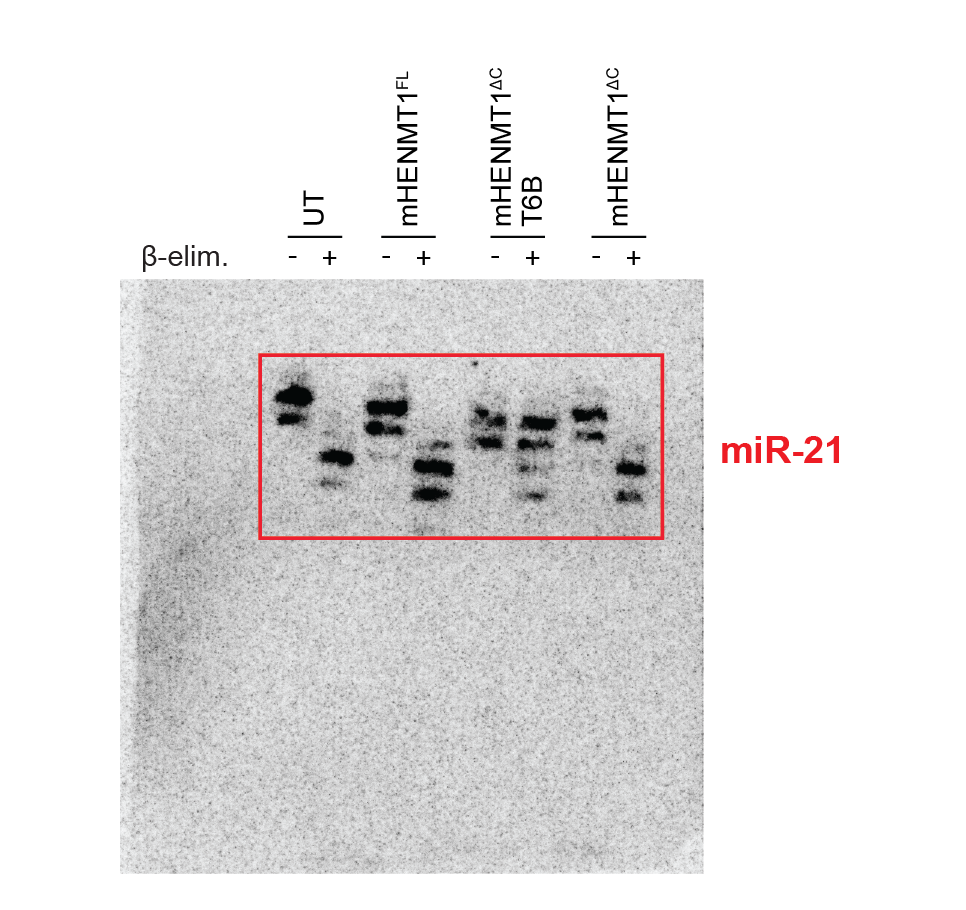

Supplement: Supplementary file 5 — Source data Fig. 1 [file 44318_2024_102_MOESM5_ESM.zip › Source data - Figure 1/1D/miR-21.png]

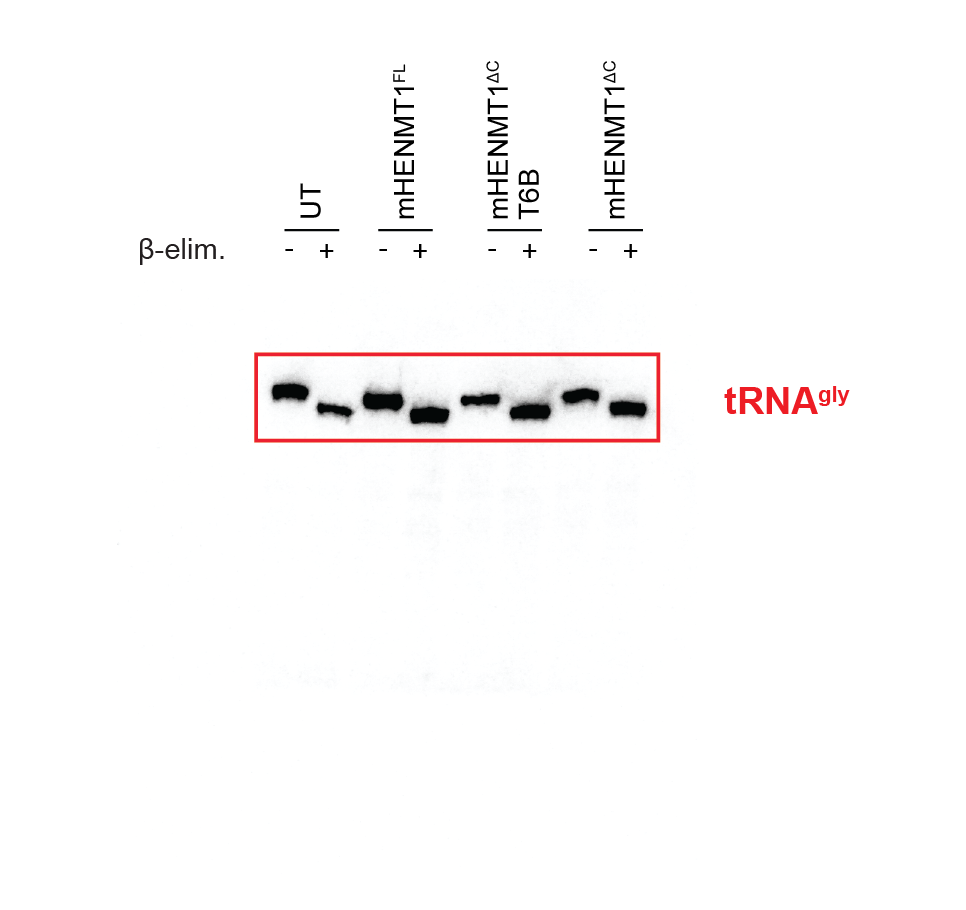

Supplement: Supplementary file 5 — Source data Fig. 1 [file 44318_2024_102_MOESM5_ESM.zip › Source data - Figure 1/1D/tRNAgly.png]

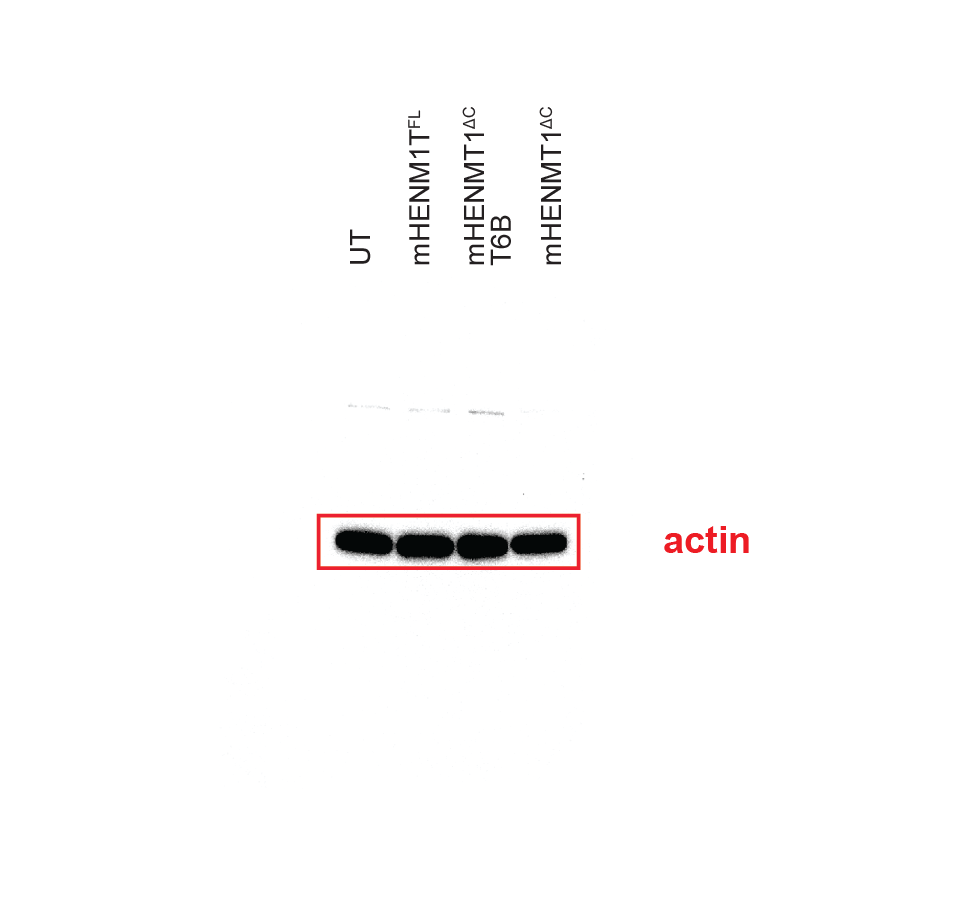

Supplement: Supplementary file 5 — Source data Fig. 1 [file 44318_2024_102_MOESM5_ESM.zip › Source data - Figure 1/1E/actin.png]

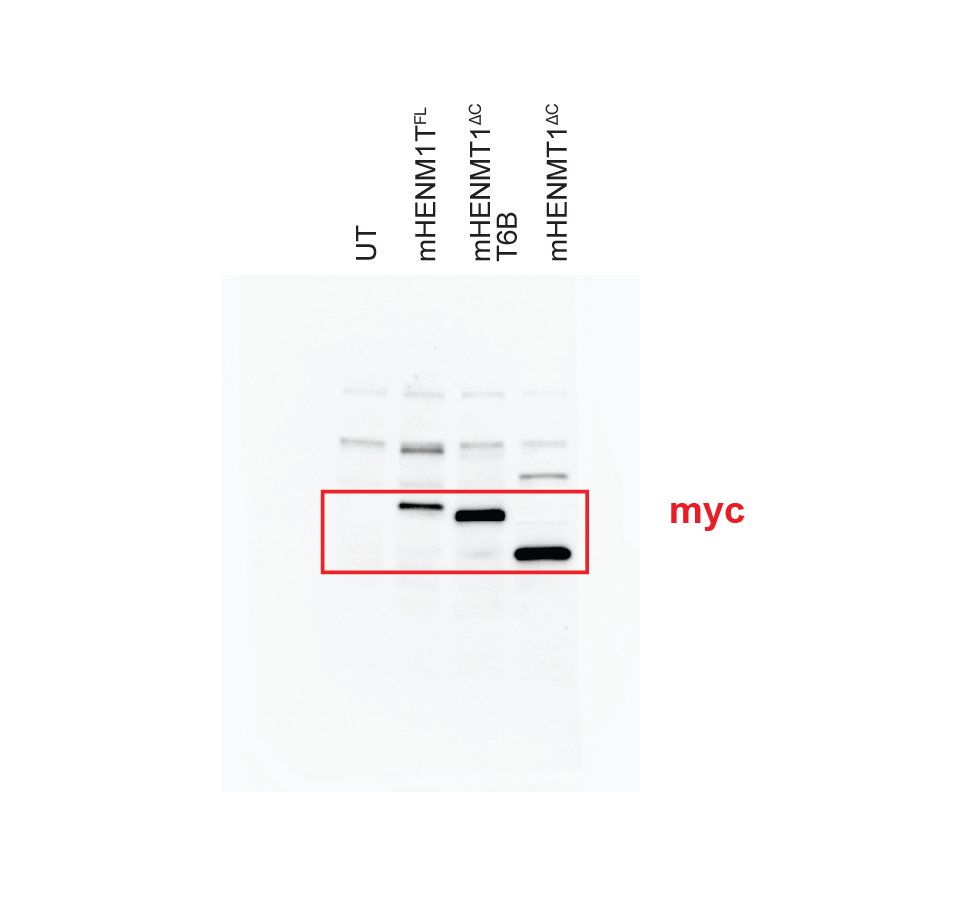

Supplement: Supplementary file 5 — Source data Fig. 1 [file 44318_2024_102_MOESM5_ESM.zip › Source data - Figure 1/1E/myc.png]
