# Supplementary material for: Mime-seq 2.0: a method to sequence microRNAs from specific mouse cell types
Source: EMBO J. 2024 Apr 30;43(12):10. doi: 10.1038/s44318-024-00102-8 (PMC11183118; doi:10.1038/s44318-024-00102-8)
Supplement: Supplementary file 7 — EV Figure Source data [file 44318_2024_102_MOESM7_ESM.zip › Source Data for Expanded View/116314_SourceDataForFigEV1.pdf]

Fig EV1A

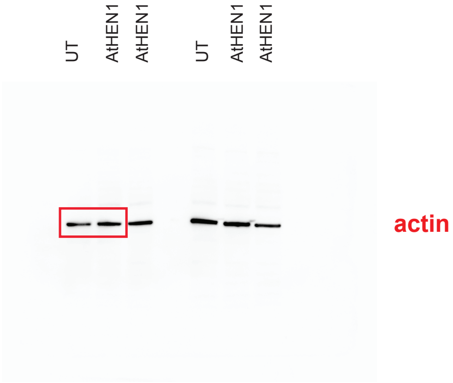

Fig EV1A

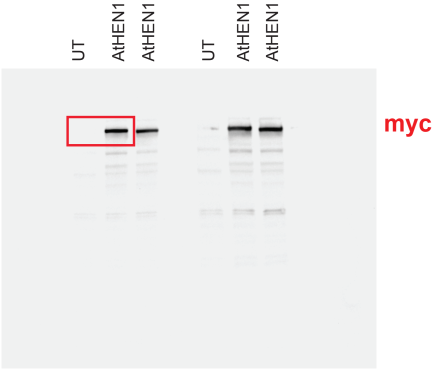

Fig EV1B

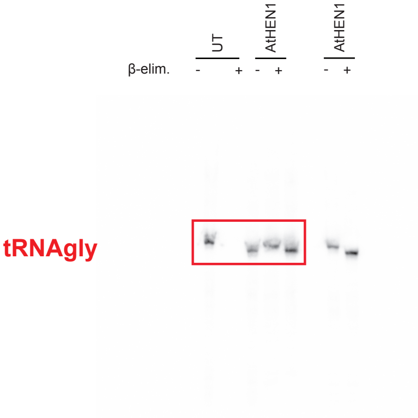

Fig EV1B

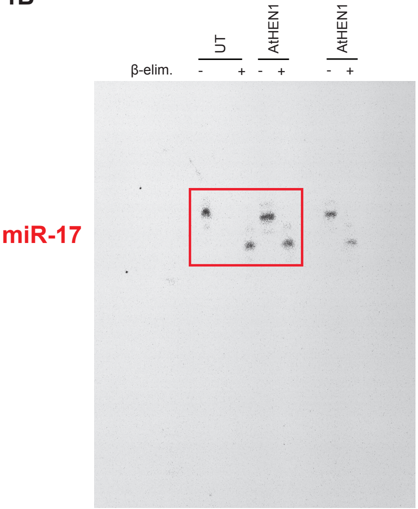

Fig EV1B

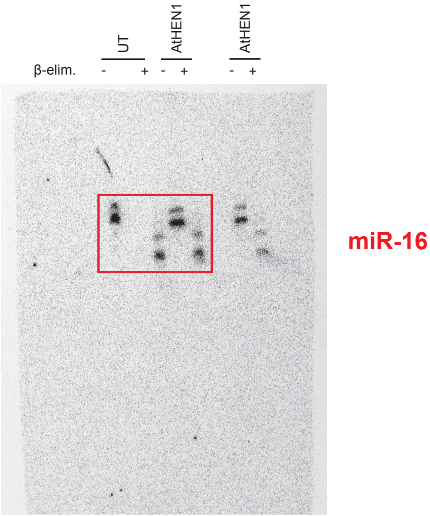

Fig EV1C

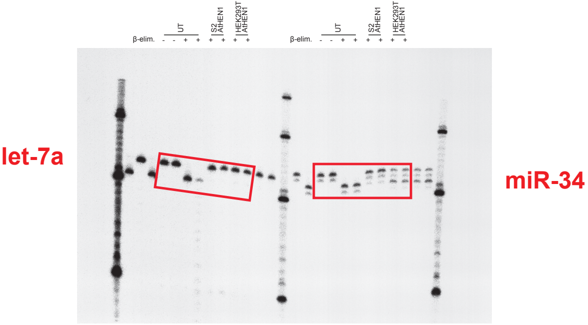

Fig EV1D

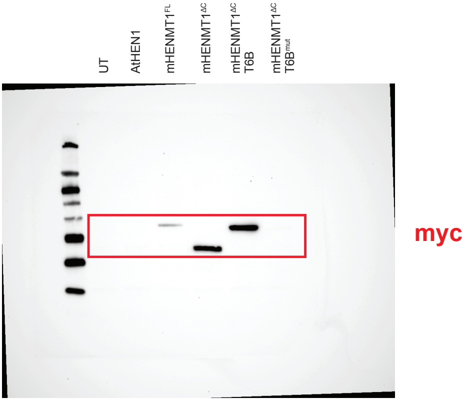
UT
AtHEN1
mHENMT1FL
mHENMT1ΔC
mHENMT1ΔC T6B
mHENMT1ΔC T6Bmut
myc

Fig EV1D

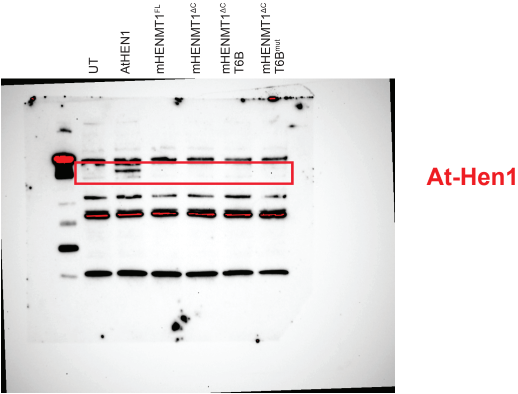
UT
AtHEN1
mHENMT1FL
mHENMT1ΔC
mHENMT1ΔC T6B
mHENMT1ΔC T6Bmut
At-Hen1

Fig EV1D

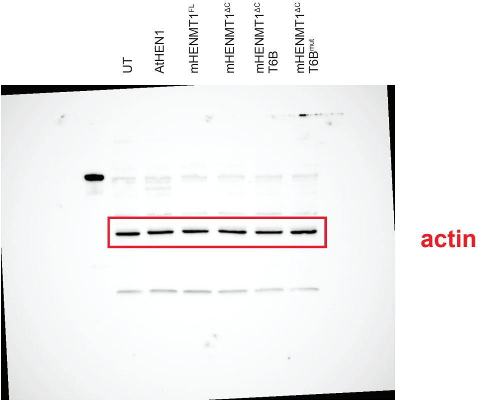

UT
AtHEN1
mHENMT1FL
mHENMT1ΔC
mHENMT1ΔC T6B
mHENMT1ΔC T6Bmut
actin

Fig EV1E

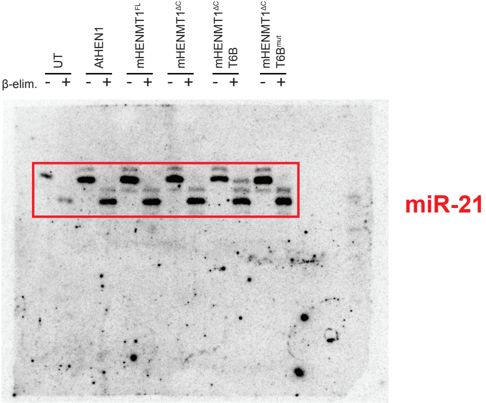
UT
AtHEN1
mHENMT1FL
mHENMT1ΔC
mHENMT1ΔC T6B
mHENMT1ΔC T6Bmut
β-elim.
- + - + - + - + - + - +
miR-21

Fig EV1E

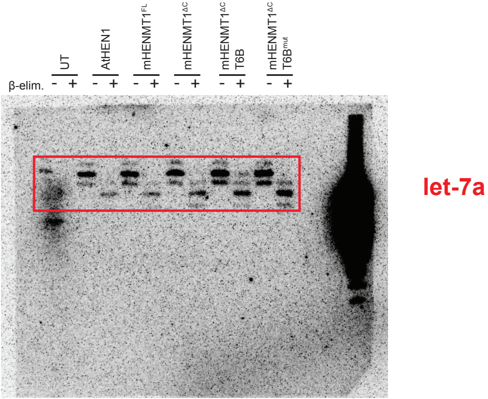
UT
AtHEN1
mHENMT1FL
mHENMT1ΔC
mHENMT1ΔC T6B
mHENMT1ΔC T6Bmut
β-elim.
- +
- +
- +
- +
- +
- +
let-7a

Fig EV1E

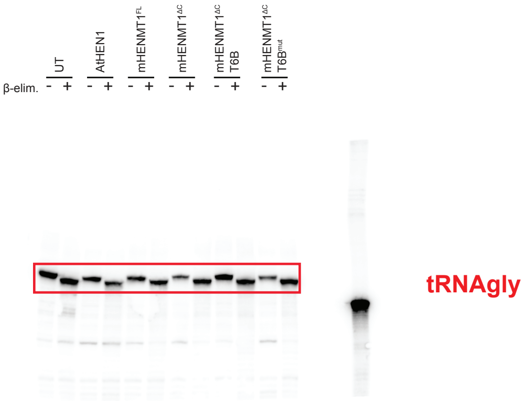
UT
AtHEN1
mHENMT1^{FL}
mHENMT1$^{\Delta C}$
mHENMT1$^{\Delta C}$ T6B
mHENMT1$^{\Delta C}$ T6B^{mut}
β-elim.
- + - + - + - + - + - +
tRNAgly

Fig EV1F

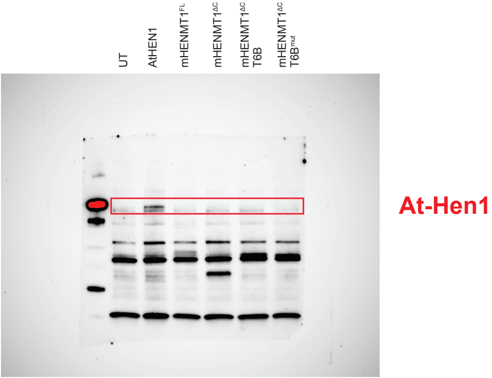
UT
AtHEN1
mHENMT1FL
mHENMT1ΔC
mHENMT1ΔC T6B
mHENMT1ΔC T6Bmut
At-Hen1

Fig EV1F

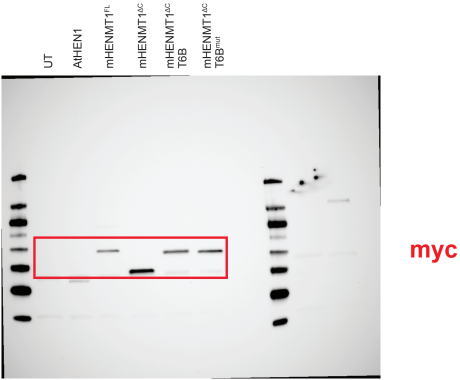
UT
AtHEN1
mHENMT1FL
mHENMT1ΔC
mHENMT1ΔC T6B
mHENMT1ΔC T6Bmut
myc

Fig EV1F

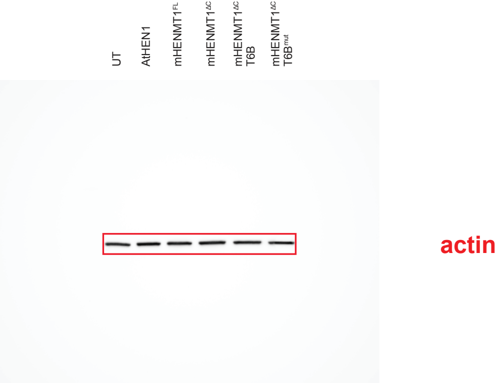
UT
AtHEN1
mHENMT1FL
mHENMT1ΔC
mHENMT1ΔC T6B
mHENMT1ΔC T6Bmut
actin

Fig EV1G

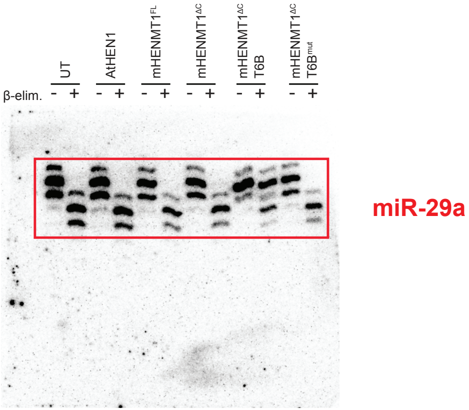
UT
AtHEN1
mHENMT1FL
mHENMT1ΔC
mHENMT1ΔC T6B
mHENMT1ΔC T6Bmut
β-elim.
- + - + - + - + - + - +
miR-29a

Fig EV1G

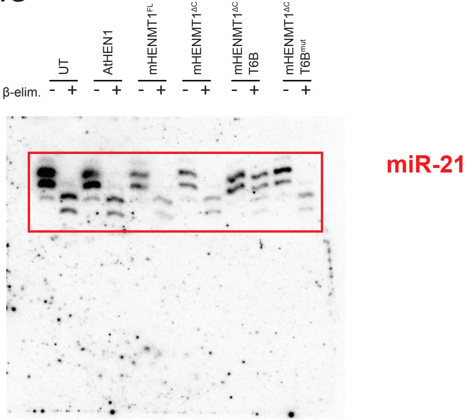
UT
AtHEN1
mHENMT1FL
mHENMT1ΔC
mHENMT1ΔC T6B
mHENMT1ΔC T6Bmut
β-elim.
- + - + - + - + - + - +
miR-21

Fig EV1G

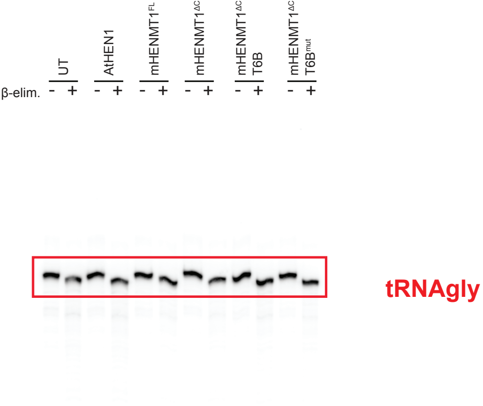
UT
AtHEN1
mHENMT1FL
mHENMT1ΔC
mHENMT1ΔC T6B
mHENMT1ΔC T6Bmut
β-elim.
- + - + - + - + - + - +
tRNAgly
