# Supplementary material for: Mime-seq 2.0: a method to sequence microRNAs from specific mouse cell types
Source: EMBO J. 2024 Apr 30;43(12):10. doi: 10.1038/s44318-024-00102-8 (PMC11183118; doi:10.1038/s44318-024-00102-8)
Supplement: Supplementary file 9 — Expanded View Figures [file 44318_2024_102_MOESM9_ESM.pdf]

Expanded View Figures

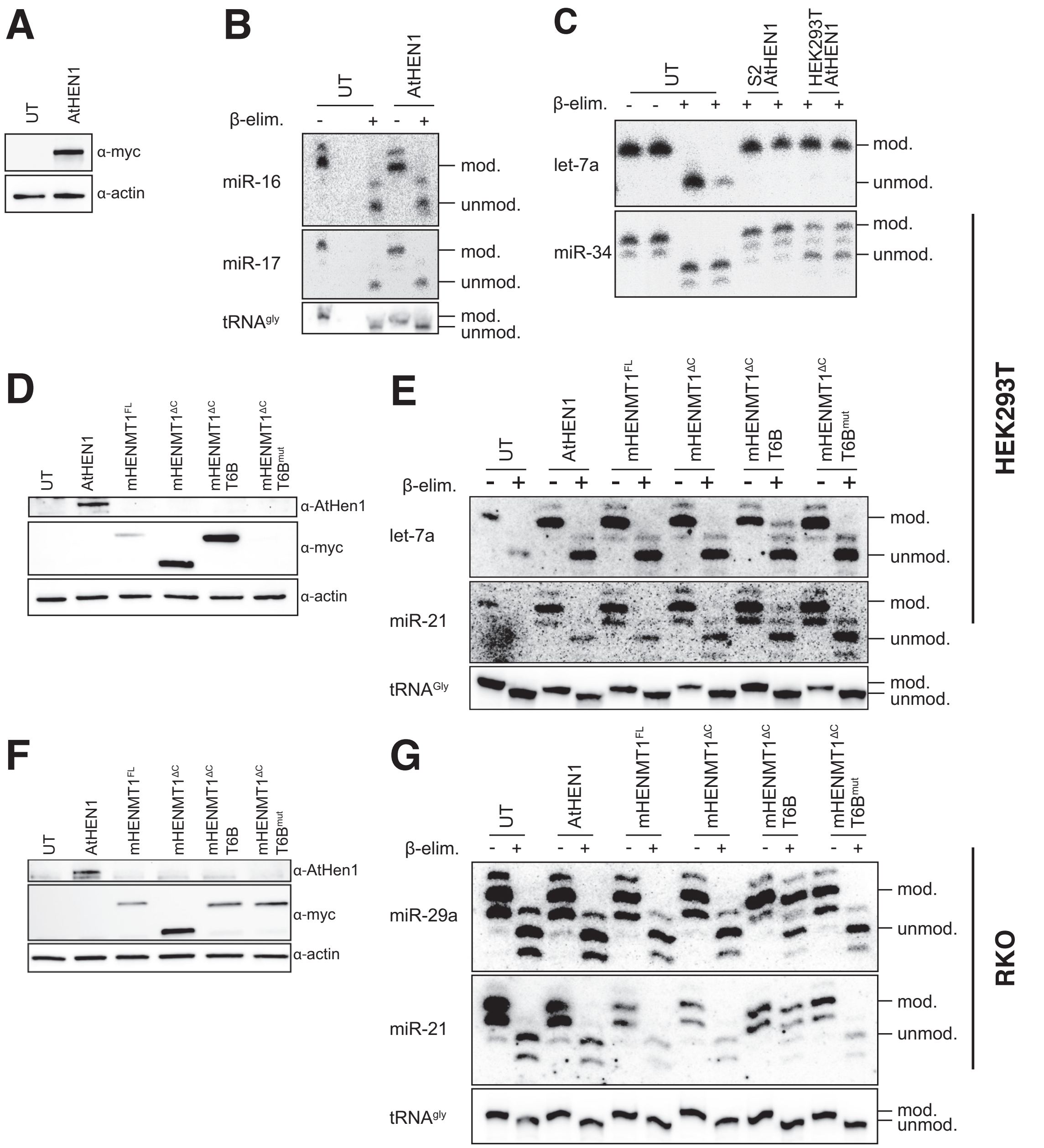

Figure EV1. HENMT1$^{\Delta C}$-T6B but not At-HEN1 efficiently methylates mouse and human miRNAs in cultured cells.

(**A–E**) HEK293T cells transfected with a construct for expression of codon optimized and Myc-tagged At-HEN1. (**A**) Western blot for Myc-tagged At-HEN1 from HEK293T cells. Actin served as loading control. (**B**) Total RNA from the HEK293T cells was subjected to oxidation and β-elimination and the products resolved by high-resolution northern blotting with the indicated probes (tRNA^{Gly} was used to monitor oxidation completion, and as loading control). (**C**) In vitro methylation assay. FLAG-tagged At-HEN1 was immunopurified upon expression in S2 cells, or Myc-tagged At-HEN1 from HEK293T cells and incubated at 37 °C with radiolabeled dme-let-7 or dme-miR-34 duplex RNAs with 2-nt 3′ overhangs. Incubation without enzyme served as negative control. Methylation was assessed by β-elimination and high-resolution PAGE. (**D**) Western blots to monitor expression of the various enzymes in HEK293T cells transfected with indicated constructs. (**E**) Total RNA extracted from HEK293T cells transiently transfected with indicated constructs, and untransfected control (UT) was treated as in Fig. 1D. (**F**, **G**) RKO cells transduced with indicated constructs under a doxycycline inducible TRE3 in cells that have integrated the rtTA3 transactivator. (**F**) Western blots to monitor expression of the various enzymes with titrated doxycycline concentrations. (**G**) Northern blots as described in (**B**). Experiments were performed at least twice per cell line. Source data are available online for this figure.

 © The Author(s)

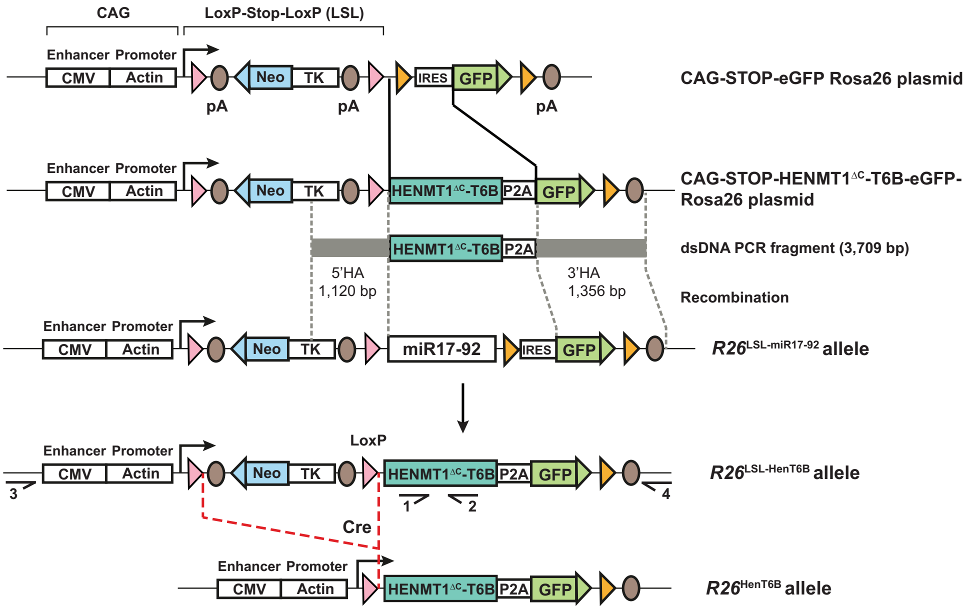

Figure EV2. Generation of the *R26*$^{LSL\text{-}HenT6B}$ allele.

The HENMT1$^{\Delta C}$-T6B cDNA was cloned in the CAG-STOP-eGFP-Rosa26 TV plasmid by replacing the IRES sequence with the HENMT1$^{\Delta C}$-T6B cDNA fused in frame via a P2A peptide to the eGFP coding sequence to generate the CAG-STOP-HENMT1$^{\Delta C}$-T6B-eGFP-Rosa26 plasmid. A 3,709-bp PCR fragment containing a 1120-bp 5′ homology arm (HA) and a 1356-bp 3′ HA was used as double-stranded DNA repair template together with two sgRNAs to replace the miR17-92-IRES DNA sequences of the *R26*$^{LSL\text{-}miR17\text{-}92}$ allele[26] by CRISPR/Cas9-mediated genome editing in mouse 2-cell embryos, resulting in the *R26*$^{LSL\text{-}HenT6B}$ allele. Cre-mediated deletion of the loxP-Stop-loxP (LSL) cassette leads to the expression of the HENMT1$^{\Delta C}$-T6B-P2A-eGFP gene from the ubiquitously transcribed CAG promoter of the *R26*^{HenT6B} allele. *LoxP* and *frt* sites are indicated by red and yellow arrowheads, respectively. The herpes simplex virus thymidine kinase (TK) promoter drives expression of the neomycin (Neo) resistance gene. Arrows indicate primers 1 and 2 used for genotyping of the *R26*$^{LSL\text{-}HenT6B}$ allele and primers 3 and 4 used for genotyping of the wild-type *R26* allele (see Methods). pA, polyadenylation sequence.

© The Author(s)

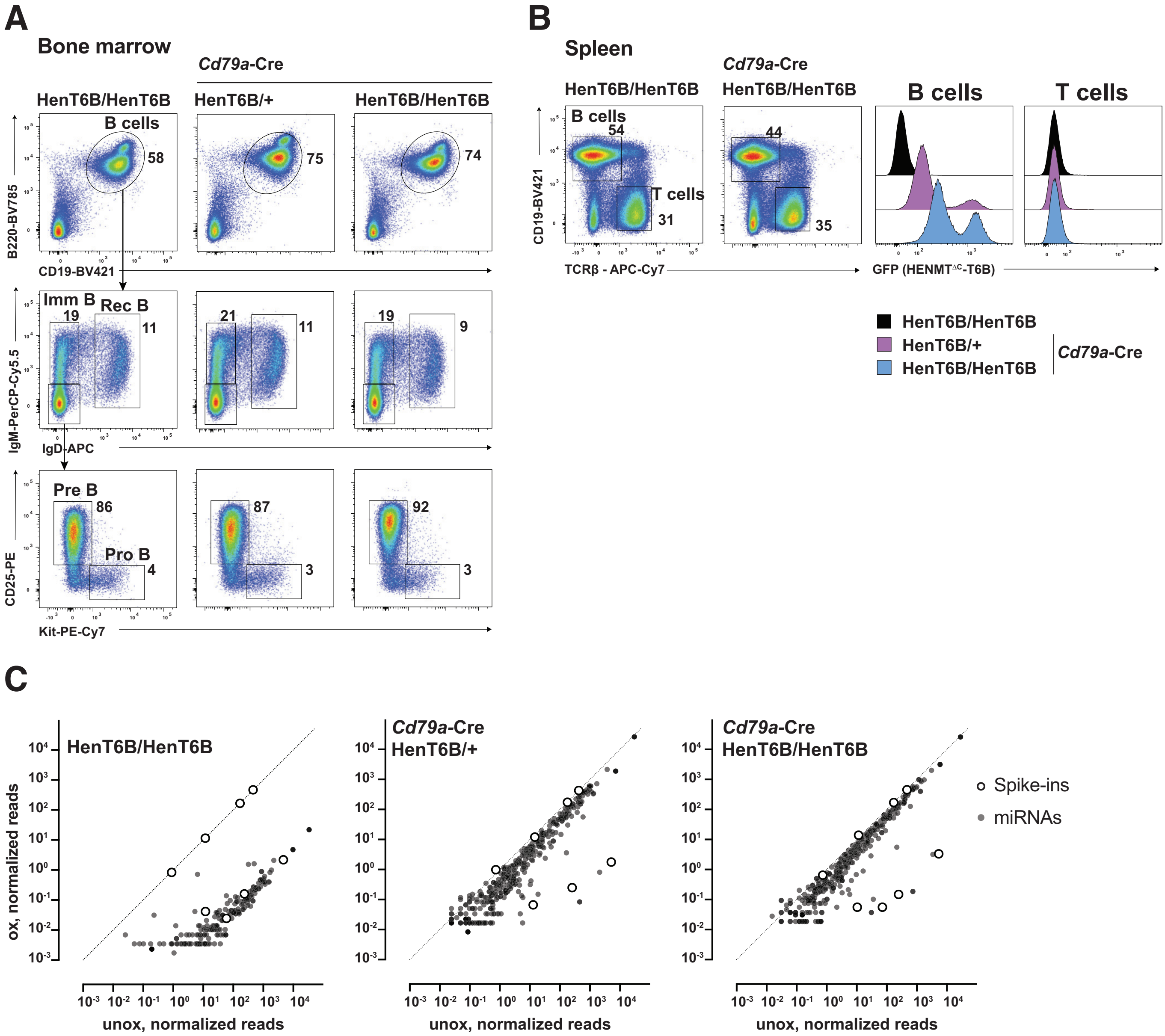

Figure EV3. Analysis of B cell development in *Cd79a*-Cre $R26^{LSL\text{-}HenT6B/LSL\text{-}HenT6B}$ mice.

(**A**) Flow-cytometric analyses of the indicated B cell types in the bone marrow of *Cd79a*-Cre $R26^{LSL\text{-}HenT6B/+}$, *Cd79a*-Cre $R26^{LSL\text{-}HenT6B/LSL\text{-}HenT6B}$ and control $R26^{LSL\text{-}HenT6B/LSL\text{-}HenT6B}$ mice at the age of 6–8 weeks. The percentage of cells in the indicated gates is shown. One of three independent experiments is shown. (**B**) Flow-cytometric analysis of B and T cells in the spleen of *Cd79a*-Cre $R26^{LSL\text{-}HenT6B/+}$, *Cd79a*-Cre $R26^{LSL\text{-}HenT6B/LSL\text{-}HenT6B}$ and control $R26^{LSL\text{-}HenT6B/+}$ or $R26^{LSL\text{-}HenT6B/LSL\text{-}HenT6B}$ mice at the age of 6–8 weeks. The percentage of B and T cells is shown next to the respective gate (left). The GFP expression of B and T cell of the indicated genotypes is shown as a histogram (right). One of three independent experiments is shown. (**C**) Total RNA was extracted from B cells of the indicated genotypes (see Methods for purification strategy), mixed with four methylated and four unmethylated RNA spike-ins and subjected to mime-seq. Libraries were normalized to methylated spike-ins. Normalized reads of oxidized vs. unoxidized libraries are plotted for every miRNA with > 0.5 norm. reads. Note the depletion of unmethylated miRNAs (similar to the four unmethylated spike-ins) and the strong enrichment when $HENMT1^{\Delta C}$-T6B is expressed (close to the four methylated spike-ins).

 © The Author(s)

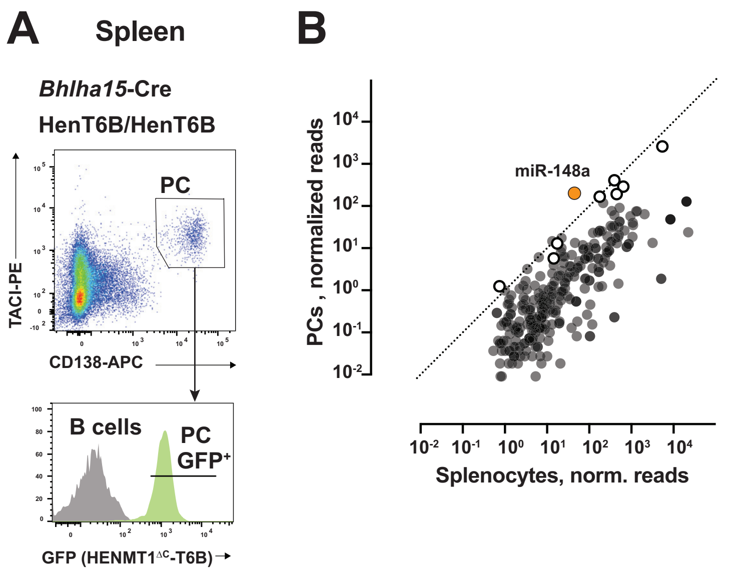

E

	100%	1%	0.1%	0.01%
total identified	85	40	33	9
false positives		0	3	7
Top 5		5	4	1
Top 10		9	6	1
Top 25		22	13	1
Top 50		36	26	1

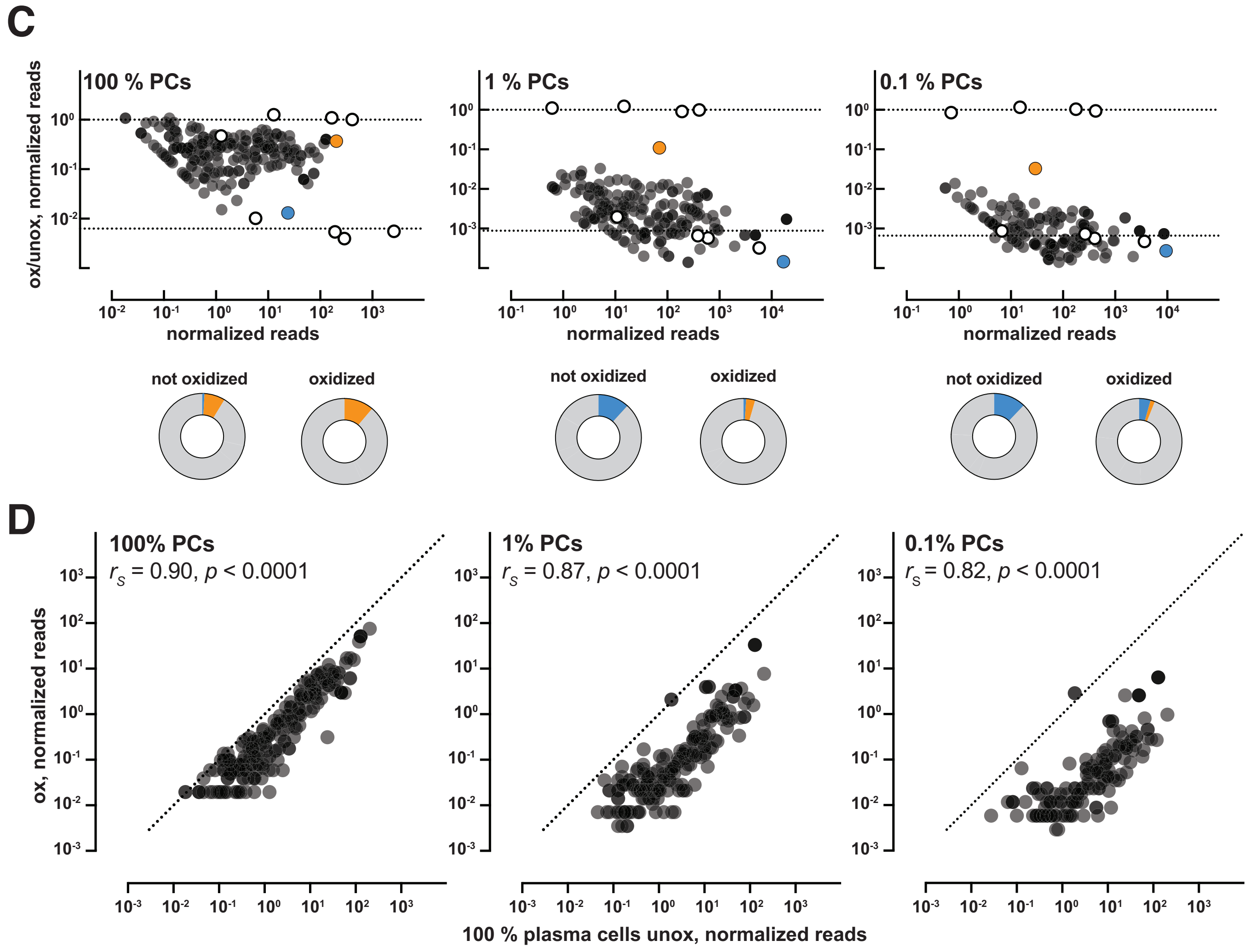

Figure EV4. Mime-seq identifies cell-specific miRNAs with high sensitivity and maintains relative abundance of miRNAs.

(**A**) Flow-cytometric sorting of plasma cells from the spleen of *Bhlha15*-Cre *R26*$^{LSL\text{-}HenT6B/LSL\text{-}HenT6B}$ mice at day 7 after immunization with sheep red blood cells. The sorting gates used for the isolation of plasma cells (CD138^{+}TACI^{+}) are indicated. (**B**) Comparison of unoxidized, spike-in normalized small RNA libraries from 100% PCs vs 99.99% splenocytes (0.01% PCs) showed that miR-148a is exclusively expressed in PCs. (**C**) Fractions recovered for all sequenced miRNAs with normalized reads >0.5 (PC mixing ratios indicated above). Pie charts below show proportions of miRNA reads from oxidized and unoxidized samples, highlighting the enrichment of miR-148 (orange) upon oxidation even from samples with 0.1% PCs, as well as the depletion of miR-451 (blue) from contaminating erythrocytes. (**D**) Oxidized libraries from samples with the indicated mixing ratios vs. normalized reads from unoxidized pure PCs. The relative abundance of miRNAs present in PCs is maintained after oxidation, showing that mime-seq remains semi-quantitative down to 0.1% PC (r_S = Spearman correlation coefficient). (**E**) Summary table of the most highly abundant PC miRNAs recovered in 1%, 0.1%, or 0.01% PCs.

© The Author(s)
